# Quantitative Visualization of the Interaction between Complement Component C1 and Immunoglobulin G: The Effect of C_H_1 Domain Deletion

**DOI:** 10.3390/ijms23042090

**Published:** 2022-02-14

**Authors:** Saeko Yanaka, Shigetaka Nishiguchi, Rina Yogo, Hiroki Watanabe, Jiana Shen, Hirokazu Yagi, Takayuki Uchihashi, Koichi Kato

**Affiliations:** 1 Exploratory Research Center on Life and Living Systems (ExCELLS), Institute for Molecular Science (IMS), National Institutes of Natural Sciences, 5-1 Higashiyama, Myodaiji, Okazaki 444-8787, Japan; saeko-yanaka@ims.ac.jp (S.Y.); shigetaka-nishiguchi@ims.ac.jp (S.N.); yogo@ims.ac.jp (R.Y.); hwatanabe0205@gmail.com (H.W.); skana@ims.ac.jp (J.S.); 2 Faculty and Graduate School of Pharmaceutical Sciences, Nagoya City University, 3-1 Tanabe-dori, Mizuho-ku, Nagoya 467-8603, Japan; hyagi@phar.nagoya-cu.ac.jp; 3 Department of Physics, Nagoya University, Furo-cho, Chikusa-ku, Nagoya 464-8602, Japan

**Keywords:** immunoglobulin G, complement component C1, high-speed atomic force microscopy, C_H_1, C_L_

## Abstract

Immunoglobulin G (IgG) adopts a modular multidomain structure that mediates antigen recognition and effector functions, such as complement-dependent cytotoxicity. IgG molecules are self-assembled into a hexameric ring on antigen-containing membranes, recruiting the complement component C1q. In order to provide deeper insights into the initial step of the complement pathway, we report a high-speed atomic force microscopy study for the quantitative visualization of the interaction between mouse IgG and the C1 complex composed of C1q, C1r, and C1s. The results showed that the C1q in the C1 complex is restricted regarding internal motion, and that it has a stronger binding affinity for on-membrane IgG2b assemblages than C1q alone, presumably because of the lower conformational entropy loss upon binding. Furthermore, we visualized a 1:1 stoichiometric interaction between C1/C1q and an IgG2a variant that lacks the entire C_H_1 domain in the absence of an antigen. In addition to the canonical C1q-binding site on Fc, their interactions are mediated through a secondary site on the C_L_ domain that is cryptic in the presence of the C_H_1 domain. Our findings offer clues for novel-modality therapeutic antibodies.

## 1. Introduction

Immunoglobulin G (IgG) is a crucial mediator of the defensive mechanisms that eliminate infectious microorganisms. Host IgG antibodies recognize antigenic determinants on the surface of invasive cells, triggering effector functions, such as cytotoxicity and opsonic phagocytosis [1]. IgG molecules adopt a modular multidomain structure constituted of two identical heavy chains and two identical light chains. The heavy chain comprises V_H_, C_H_1, C_H_2, and C_H_3 domains, whereas the light chains are divided into V_L_ and C_L_ domains. One IgG molecule can be separated into two Fab and one Fc fragments, tethered at a flexible, disulfide-linked hinge region connecting the C_H_1 and C_H_2 domains. Antigen recognition is carried by the two Fab portions, each composed of V_H_, V_L_, C_H_1, and C_L_ domains. Consequently, effector functions are promoted through the Fc region, comprising a pair of C_H_2–C_H_3 segments as a twofold symmetrical dimer.

A variety of IgG molecules are currently being used as therapeutic antibodies because of their antigen-binding specificities and/or cytotoxic ability [2,3]. The cytotoxicity of IgG is mediated by the first complement component, C1, or receptors for the IgG–Fc portion, which are collectively termed Fcγ receptors (FcγRs) [4,5]. IgG binds these effector molecules primarily through its hinge-proximal region spanning the two C_H_2 domains. The conformational and functional integrity of this canonical binding site is maintained and regulated by hinge disulfide bridges and a pair of Asn297-linked glycans [6,7,8]. Furthermore, protein engineering approaches have been applied by targeting this site in order to improve the affinities for the effector molecules and the consequent efficacy of therapeutic antibodies [9].

A long-standing question regarding the way in which antigen recognition by the Fab region triggers the effector functions evoked by the Fc region remains unresolved [10]. In addition to the canonical binding site, other interaction sites for effector molecules are built into the IgG molecule, as exemplified by an additional subsite in the Fab region of human IgG1 for interaction with FcγRIII [11,12]. Antigen binding may impact the conformations of the secondary binding site, thereby allosterically affecting the Fab–FcγRIII interaction. Such non-canonical binding sites are potential targets for the engineering of the higher functionality of therapeutic antibodies.

Another mechanism is the assembly of antigen-bound IgG molecules, facilitating their multivalent interactions with effector molecules. Indeed, IgG molecules are self-assembled into a hexameric ring on antigen-containing membranes, recruiting C1q, which is a subcomponent of the first component of the classical complement pathway [13,14]. The hexamer formation of human IgG1 is mediated through the interfacial region between the C_H_2 and C_H_3 domains, and can be enhanced by mutational modification at the region, which therefore can be a target for the improvement of the complement-dependent cytotoxicity (CDC) of therapeutic antibodies [15,16].

We have established a method for the quantitative visualization of IgG interactions with C1q and FcγRIII by high-speed atomic force microscopy (HS-AFM) [11,13]. Here we apply this method to characterize the interaction between IgG and the C1 complex, comprising C1q, C1r, and C1s. Furthermore, besides intact IgGs, we performed HS-AFM on a unique IgG variant that lacks the entire C_H_1 domain and can activate the complement pathway even without antigen [17]. Our observations will provide dynamic views of the molecular process at the initial step of the complement pathway, and clues for antibody engineering to control CDC activity.

## 2. Results and Discussion

### 2.1. Comparing the Conformational Flexibility between C1 and C1q

**Figure 1:**
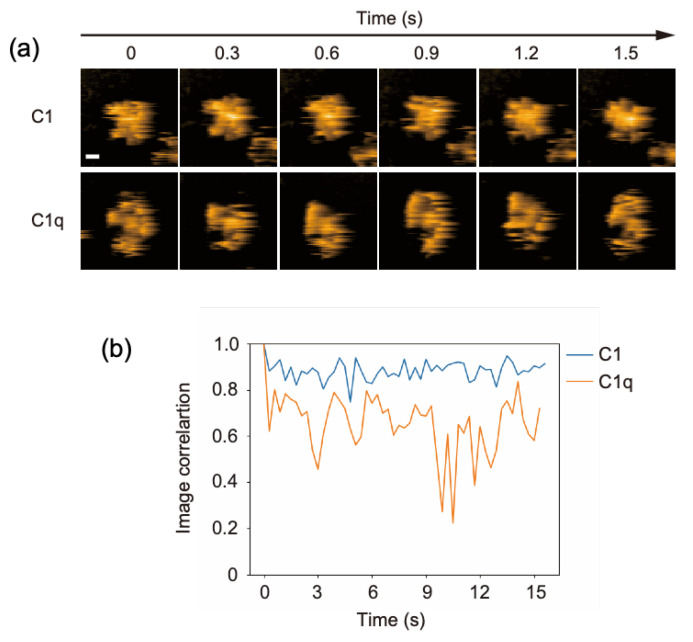
HS-AFM observation of C1 and C1q. (**a**) Clipped AFM images of C1 and C1q observed on the mica surface. Scale bar = 20 nm. (**b**) Time courses of the image correlation coefficient for C1 and C1q. The image correlation coefficient for each frame was calculated between the corresponding frame and the previous frame [19]. The larger fluctuation of the correlation coefficient for C1q than for C1 suggests that C1q has more structural flexibility.

C1q is a 400-kDa protein constituted from 18 polypeptides assembled into six globular domains tethered to a central stem with a collagen-like structure. It associates with two C1r and two C1s subunits, forming the C1 complex. In our previous HS-AFM study, the dynamic structures of free C1q molecules on a mica surface were visualized [13]. This was confirmed in the present study: its six globular heads exhibited high mobility, randomly fluctuating around the stem. In contrast, the C1 complex seemed to have a more rigid structure harboring a central mass corresponding to C1r and C1s subunits (Figure 1a, Supplementary Movies S1 and S2). In order to compare the structural flexibility of C1 and C1q, we analyzed the image correlation, allowing us to evaluate the similarity of the two images (Figure 1b). Here, for C1 or C1q, the image correlations were calculated for two consecutive frames. The closer the correlation coefficient is to 1, the higher the similarity between the two images, i.e., the less structural flexibility there is. These data indicate that the C1r and C1s subunits are associated with the central part of C1q, restraining its internal motion. Our HS-AFM observation agrees with early negative stain electron microscopy data showing that the distribution of the C1q spoke angle is restricted by C1r and C1s [18]; moreover, it provides dynamic views of C1q in complexed and uncomplexed states.

### 2.2. Comparing Dynamic Interactions of IgG Assemblages with C1 and C1q on Antigen-Incorporated Membranes

**Figure 2:**
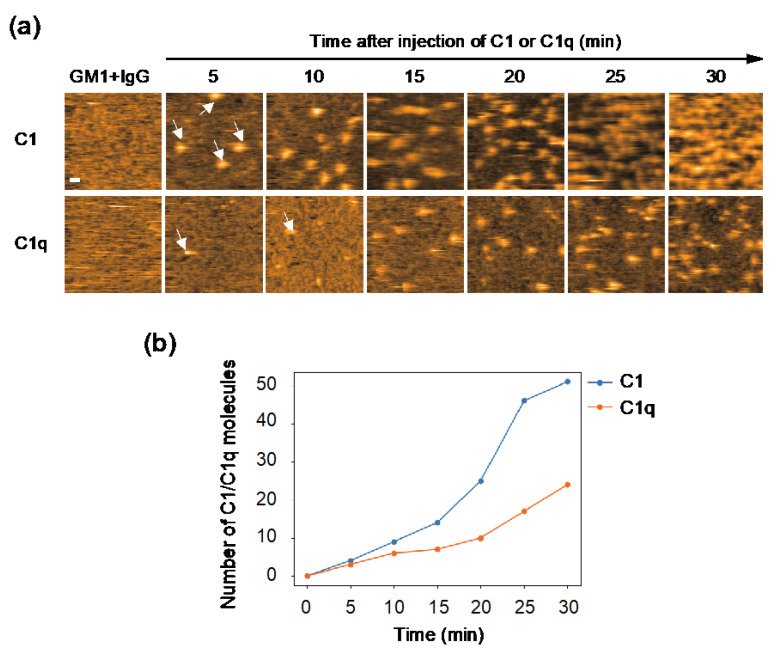
HS-AFM observation of C1/C1q interaction with IgG assemblages on antigen-incorporated membranes. (**a**) HS-AFM images every 5 minutes, showing the interaction of C1/C1q with the anti-GM1 antibody assembling on DOPC membranes containing 50% GM1. Typical images showing C1/C1q bound to the IgG assemblages (indicated by the white arrows). Scale bar = 20 nm. (**b**) The amount of C1/C1q residing on the IgG assemblages formed on the GM1-incorporated membrane, increasing depending on time, was quantified.

The observed difference in internal motion between C1 and C1q may affect their interactions with IgG. In order to address this issue, we quantified their IgG-binding affinities using GB2, a mouse monoclonal IgG2b antibody which is directed against *Campylobacter jejuni* and cross-reacts with GM1 ganglioside [20]. Our previous HS-AFM study showed that GB2 antibodies assemble into hexameric rings on GM1-containing membranes, and thereby recruit C1q [13]. Here, we compared the recruitment extent onto the IgG assemblages on the antigen-incorporated membranes between C1 and C1q. The results indicate a significantly higher amount of C1 accumulated on the IgG-covered membranes than C1q (Figure 2), explaining the slower off-rate of C1 than C1q on the IgG-immobilized surface shown by the previous surface plasmon resonance experiment [21]. It is supposed that the binding of the IgG hexameric ring suppresses the motional freedom of the C1q globular heads. This conformational entropy loss is less pronounced in C1q complexed with C1r and C1s, which may explain its higher affinity than C1q alone.

### 2.3. C1/C1q Interaction with IgG2a(s)

**Figure 3:**
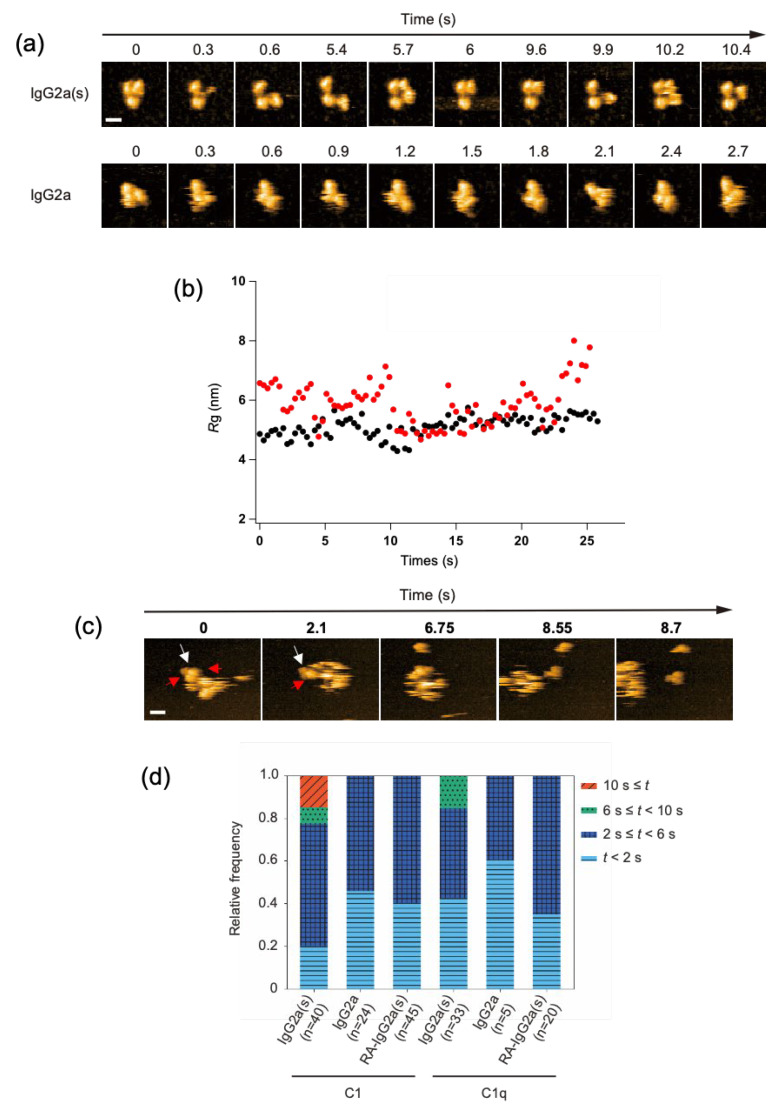
HS-AFM observation of IgG2a(s) and IgG2a, and their interactions with C1/C1q. (**a**) Clipped HS-AFM images of IgG2a and IgG2a(s) observed on the mica surface. Scale bar = 10 nm. (**b**) The *R*g value was calculated for IgG2a (black) and IgG2a(s) (red) as the average distance between the center of mass and the globular domains, as described previously [26]. (**c**) The interaction of IgG2a(s) and C1q was observed at the single-molecule level. The white arrow indicates IgG2a(s), whereas the red arrow indicates the C1q head binding to IgG2a(s). Scale bar = 20 nm. (**d**) The dwell times of C1/C1q on IgG2a, IgG2a(s), and reduced and alkylated IgG2a(s) (RA-IgG2a(s)). The relative frequency of C1/C1q observed during a given window of the dwell time (*t*) on different antibodies.

We investigated the potential impact of C_H_1 domain deletion on the structure and C1/C1q-interactions of IgG at the single-molecule level. We employed the anti-dansyl mouse IgG2a variant with shorter heavy chains devoid of the C_H_1 domain (Supplementary Figure S1) because of its ability to bind C1q and thereby activate complements under antigen-free conditions [17]. Hereafter, this *short-chain* IgG2a(κ) variant will be designated as IgG2a(s), whereas its full-length counterpart will be referred to simply as IgG2a. HS-AFM showed that the IgG2a(s) variant was monomeric and exhibited a more extended conformation with a gyration radius (*R*g) of 59 ± 0.73 nm than IgG2a (*R*g, 51 ± 0.33 nm). Previous small-angle X-ray scattering data also indicated an extended molecular shape of IgG2a(s) compared with IgG2a [22] (Figure 3a,b, and Supplementary Movies S3 and S4).

We examined the possible binding of the complement components to these sparsely distributed IgG molecules on the mica surface. Whereas C1 and C1q interacted with IgG2a only transiently, with residence times less than 6 s, they stayed on IgG2a(s) for significantly longer times. Notably, C1 had a longer residence time than C1q, often remaining on the monomeric IgG2a(s) molecule for more than 10 s (Figure 3c,d, and Supplementary Movies S5 and S6). The observed high affinities of IgG2a(s) for C1/C1q were compromised by the cleavage of its inter-chain disulfide bridges. This agrees with the previous report showing that the reduction-alkylation of hinge disulfides leads to reduced binding toward C1/C1q [17].

The 1:1 stoichiometric interaction between IgG2a(s) and C1/C1q, as confirmed by the single-molecule HS-AFM observations (Figure 3c, and Supplementary Movie S6), excludes the possibility that the enhanced complement-binding affinity of IgG2a(s) is due to its aggregation or oligomerization. Alternative explanations include the possibility that C_H_1 deletion results in the conformational activation of the canonical C1q-binding site on Fc and/or the exposure of a secondary binding site. Our ^13^C-NMR studies detected no conformational alteration of Fc on the C_H_1 domain deletion, supporting the latter possibility [23,24,25]. Indeed, high-resolution HS-AFM data of IgG2a(s) interacting with C1q showed that more than one globular head of C1q could simultaneously bind one IgG2a(s) molecule.

**Figure 4:**
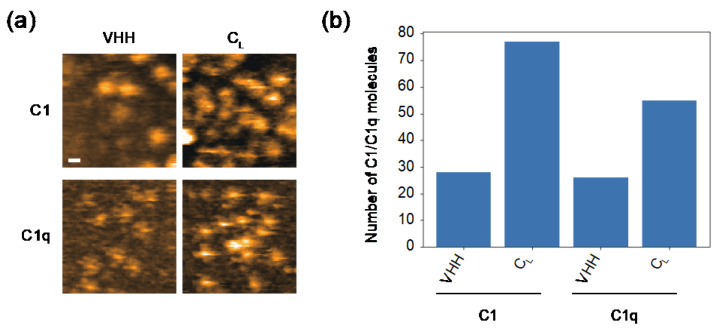
HS-AFM observation of the interaction of C_L_/VHH with C1/C1q. (**a**) Typical HS-AFM images of C1 and C1q observed on C_L_- or VHH-covered mica surfaces. Scale bar = 20 nm. (**b**) The number of C1/C1q observed on the C_L_- or VHH-covered mica surface.

Because the surfaces of the V_H_ and C_L_ domains are exposed by C_H_1 deletion [25,27], the cryptic C1q-binding site is likely to locate there. In order to test this, we examined the possible interactions of these domains with C1/C1q. For HS-AFM observation, a mouse C_L_(κ) domain with a C-terminal hexahistidine tag was immobilized on a Ni^2+^-coated mica surface. Because V_H_ domains are generally insoluble [28], we employed an anti-lysozyme VHH domain instead. Intriguingly, C1 and C1q preferentially bound C_L_ rather than VHH (Figure 4). As in the case of hexameric IgG, C1 accumulated more on the C_L_-covered mica surface than C1q. We also performed a HS-AFM-based competition experiment using C_L_ and VHH against the complement binding to the IgG assemblages. The results indicated that C_L_ had a higher inhibitory activity than VHH (Supplementary Figure S2). These data indicate that the C_L_ domain provides the secondary C1q-binding site, enabling a single IgG2a(s) molecule to undergo multivalent binding to C1q, activating the classical complement pathway in the absence of an antigen.

In 1993, Mizutani et al. hypothesized that mouse C_L_(κ) harbors a potential C1q-binding motif comprising Lys147, Lys149, and Asp151, which—regarding spatial arrangements of positive and negative charges—resembles the C1q-binding motif in the C_H_2 domain (Glu318, Lys320, and Lys322) proposed based on site-directed mutagenesis data [17]. However, a recent cryo-electron microscopic study revealed that only Lys322 is directly involved in the interaction with C1q [16]. Hence, the cryptic C1q-binding site on the C_L_ surface needs to be revisited.

In order to improve the CDC activity of therapeutic antibodies, protein engineering approaches have been employed to enhance C1q–Fc interaction and Fc-mediated IgG hexamerization [15,29]. This study suggests that the cryptic C1q-binding site in C_L_ is an alternative target for antibody engineering to enhance the C1-binding affinity of IgG and the consequent activation of the classical complement pathway. Our future work will involve the identification of the C1q-binding motif in C_L_, and will investigate whether it can be embedded into human IgG to activate the complement pathway in its monomeric state. Such an approach will play a complementary or synergistic role to the hexamerized IgG-based approach, thereby opening up new possibilities for the development of novel-modality therapeutic antibodies.

## 3. Materials and Methods

### 3.1. Chemicals

The GM1 and DOPC were purchased from Avanti Polar (Alabaster, AL, USA).

### 3.2. Protein Preparation

#### 3.2.1. Antibody

The mouse monoclonal anti-GM1 IgG2b(κ) antibody, GB2, was produced in mouse hybridoma cells [20]. The mouse monoclonal anti-dansyl IgG2a and IgG2a(s) were produced in the switch variant cell lines 27–13.6 and 27–1B10.7, respectively [30]. The cells were cultivated in an NYSF 404 serum-free medium (Nissui, Tokyo, Japan). After the cell growth, the medium supernatant was applied to an nProtein A Sepharose Fast Flow column (GE Healthcare, Chicago, IL, USA), followed by size-exclusion chromatography using a HiLoad 16/60 Superdex 200 pg column (GE Healthcare, Chicago, IL, USA) with phosphate-buffered saline (PBS) consisting of 137 mM NaCl, 2.7 mM KCl, 8.1 mM Na_2_HPO_4_, and KH_2_PO_4_ (pH 7.4) to purify the IgG antibodies. Analytical size-exclusion chromatography confirmed that the IgG2a and IgG2a(s) preparations contained no detectable aggregate or oligomer (Supplementary Figure S3). For the cleavage of the interchain disulfide bridges, IgG2a(s) was reduced by 10 mM DTT at room temperature for 1 h in 1.5 M Tris-HCl, pH 8.5, containing 2 mM EDTA. In total, 22 mM iodoacetic acid was added to the above reaction mixture, which was incubated in the dark for 20 min at room temperature. Finally, the IgG2a(s) antibody and its reduced and alkylated analog were dialyzed against PBS, and were subjected to HS-AFM measurements.

Mouse C_L_(κ) domain with a C-terminal Cys to Ser mutation followed by a hexahistidine tag was subcloned into pET21a (Merck Millipore, Burlington, MA, USA), and was expressed in *Escherichia coli* BL21-(DE3) (Agilent Technologies, Santa Clara, CA, USA). For the recombinant C_L_(κ) domain expression, the *E. coli* cells were grown in Luria-Bertani medium containing ampicillin. After sonication and centrifugation, the soluble fraction of the cell lysate was subjected to affinity chromatography with Ni^2+^-charged Chelating Sepharose (Cytiva, Tokyo, Japan). The resultant C_L_ domain was further purified by size- exclusion chromatography using a Superdex 75 pg column (Cytiva, Tokyo, Japan). Camelid anti-lysozyme VHH domain D3-L11 with a C-terminal hexahistidine tag was prepared as described previously [31]. Analytical size-exclusion chromatography confirmed that the mutated C_L_ and VHH domains were both monomeric (Supplementary Figure S3). The conformational integrity of the C_L_ domain was confirmed based on ^1^H-^15^N heteronuclear single-quantum coherence spectral data (Supplementary Figure S4).

#### 3.2.2. C1q

The C1 was purchased from Fitzgerald Industries International, Acton, MA, USA. The C1q was purified from 40 mL pooled human serum (Cosmo Bio CO., LTD, Tokyo, Japan) via two-step precipitation at low ionic strength, as previously described [13]. The supernatant contained 0.2 mg/mL C1q.

### 3.3. HS-AFM Observation

A mica substrate with a diameter of 1.5 mm and a thickness of 0.1 mm (Furuuchi Chemical, Tokyo, Japan) was attached with glue on a glass stage. A 2 μL droplet of 0.01% (for complements) or 0.1% (for antibodies) 3-aminopropyltriethoxysilane (APTES) solution was placed on a freshly cleaved mica substrate and incubated for 3 minutes. The APTES-mica substrate was then washed twice with 80 μL milli-Q water. A 2 μL droplet of IgG2a or IgG2a(s) solution was placed on the APTES-mica substrate for 3 min, and then washed with 80 μL TNC buffer (50 mM Tris-HCl (pH 8.0), 150 mM NaCl, 2 mM CaCl_2_). In order to measure the binding time between the complements and antibodies, a 2 μL droplet of protein solution was placed on a freshly cleaved mica substrate without APTES. The concentration of the antibodies and complements for adsorption was adjusted based on the pilot observations. The C1 was incubated in TNC buffer for 5–10 min for calcium-dependent activation before it was loaded onto the mica substrate. Notably, C1 activation was performed in all of the experiments described below. All of the HS-AFM observations were performed in TNC buffer at room temperature (25 °C) using a laboratory-built HS-AFM operated in tapping mode [32,33]. Small cantilevers (BL-AC7DS: Olympus, Tokyo, Japan) with a spring constant of ~0.2 Nm^−1^, a quality factor of approximately 2, and a resonant frequency of ~0.8 MHz (all properties were estimated in water) were used. The tips of the cantilevers were sharpened by electron beam deposition and argon gas etching [26,34]. Furthermore, in order to achieve a small tip-sample loading force, the free oscillation amplitude of the cantilevers was set at 1~2 nm, and the set point of the amplitude for the feedback control was approximately 90% of the free amplitude. The correlation analysis of the complements was performed by calculating the 2D correlation coefficients between the HS-AFM images of the frame and the former frame in each frame within the region of interest [35]. The binding time between the antibodies and complements was analyzed using sequential HS-AFM images by inspecting the large bright spots (complements) bound to small bright spots (antibodies) in the HS-AFM images.

In order to measure the accumulation time of complements on the anti-GM1 IgG2b assemblages formed on the membranes, ganglioside GM1 and DOPC (GM1-DOPC) were dissolved in methanol/chloroform at a 1:1 ratio to form liposomes, as described previously. GM1-DOPC was dissolved in Milli-Q water containing 5 mM MgCl_2_ after the organic solvent was removed by drying. In total, 0.01 mg/mL of the GM1-DOPC solution was sonicated using a probe-type ultrasonic homogenizer. A 2 µL droplet of the GM1-DOPC solution was placed on a freshly cleaved mica substrate on a glass stage, and was incubated at 70 °C for 20 min in a sealed container in order to maintain a high humidity, to prevent surface drying. After the incubation, the mica substrate was washed five times with 80 μL of Milli-Q water. A 2 μL droplet of 0.03 mg/mL antibody in solution was placed on the lipid-coated mica substrate for 10 min, and then washed with 80 μL TNC buffer. The complements were added at an 18–59 μg/mL final concentration using a pipette during the HS-AFM observation. For the competition experiments, the complements were mixed with 3 molar equivalents of C_L_ or VHH domains. The number of complements in the scanning area (200 × 200 nm^2^) was counted every 5 min until 30 min after the addition of the complements.

In order to measure the number of complements on the C_L_ domain-coated mica substrate, a 2 μL droplet of 2 mM NiCl_2_ solution was placed on a freshly cleaved mica substrate. After 3 min incubation, the nickel-mica substrate was washed with 10 μL Milli-Q water. A 1 μL droplet of hexahistidine-tagged protein solution (the C_L_ domain or VHH was placed onto the mica substrate in order to immobilize the proteins by Ni^2+^-histidine chelation, and then the mica substrate was washed with 10 μL TNC buffer). A 2 μL droplet of the complements in solution was placed onto the mica substrate, and the mica substrate was washed with 10 μL TNC buffer. The number of complements in the scanning area (200 × 200 nm^2^) was counted.

## Supplementary Materials

The following supporting information can be downloaded at: https://www.mdpi.com/article/10.3390/ijms23042090/s1. Supplementary Movies S1–S6 are available online.
