# Supplementary material for: Quantitative Visualization of the Interaction between Complement Component C1 and Immunoglobulin G: The Effect of CH1 Domain Deletion
Source: Int J Mol Sci. 2022 Feb 14;23(4):2090. doi: 10.3390/ijms23042090 (PMC8876274; doi:10.3390/ijms23042090)
Supplement: Supplementary file 1 [file ijms-23-02090-s001.zip › supplementary_information/supplementary-information.pdf]

Supplementary information

Quantitative visualization of the interaction between complement component C1 and immunoglobulin G: The effect of C_H1 domain deletion

Saeko Yanaka [1,2,†], Shigetaka Nishiguchi [1,†], Rina Yogo [1,2,†,#], Hiroki Watanabe [1], Jiana Shen [1,2], Hirokazu Yagi [2], Takayuki Uchihashi [1,3,*], and Koichi Kato [1,2,*]

[1] Exploratory Research Center on Life and Living Systems (ExCELLS), and Institute for Molecular Science (IMS), National Institutes of Natural Sciences, 5-1 Higashiyama, Myodaiji, Okazaki, 444-8787, Japan; saeko-yanaka@ims.ac.jp, shigetaka-nishiguchi@ims.ac.jp, yogo@ims.ac.jp, hwatanabe0205@gmail.com, skana@ims.ac.jp

[2] Faculty and Graduate School of Pharmaceutical Sciences, Nagoya City University, 3-1 Tanabe-dori, Mizuho-ku, Nagoya, Aichi, 467-8603, Japan; hyagi@phar.nagoya-cu.ac.jp,

[3] Department of Physics, Nagoya University, Furo-cho, Chikusa-ku, Nagoya, Aichi 464-8602, Japan

† These three authors contributed equally to this work.

# Current address: Biomedical Research Centre, School of Biomedical Engineering, The University of British Columbia 2222 Health Sciences Mall, Vancouver, BC Canada V6T 1Z3

* Correspondence: uchihast@d.phys.nagoya-u.ac.jp (T.U.); kkatonmr@ims.ac.jp (K.K.)

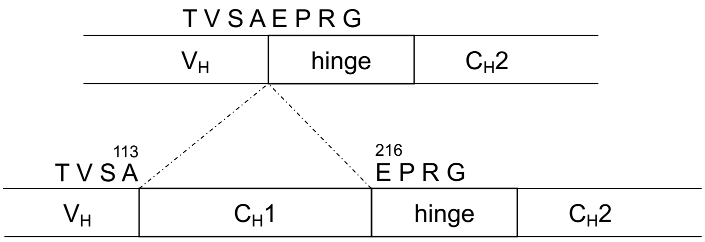

Supplementary Figure S1. Schematic drawing of C_H1 domain deletion in IgG2a(s) (adopted from reference [22]).

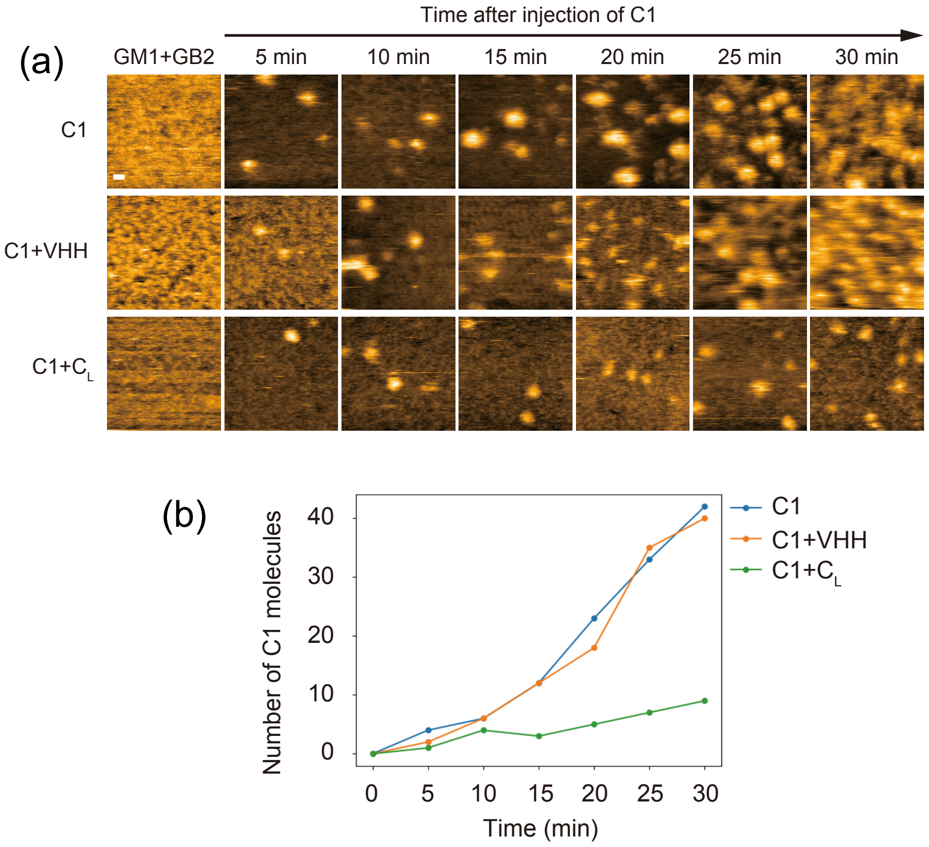

Supplementary Figure S2. HS-AFM observation of C1 interaction with IgG assemblages on antigen-incorporated membranes in the presence and absence of 3 molar equivalent of CL or VHH domains. (a) HS-AFM images every 5 min, showing the interaction of C1 with the anti-GM1 antibody assembling on DOPC membranes containing 50% GM1. Scale bar = 20 nm. (b) The number of C1 residing on the IgG assemblages formed on the GM1-incorporated membrane, increasing depending on time, was counted.

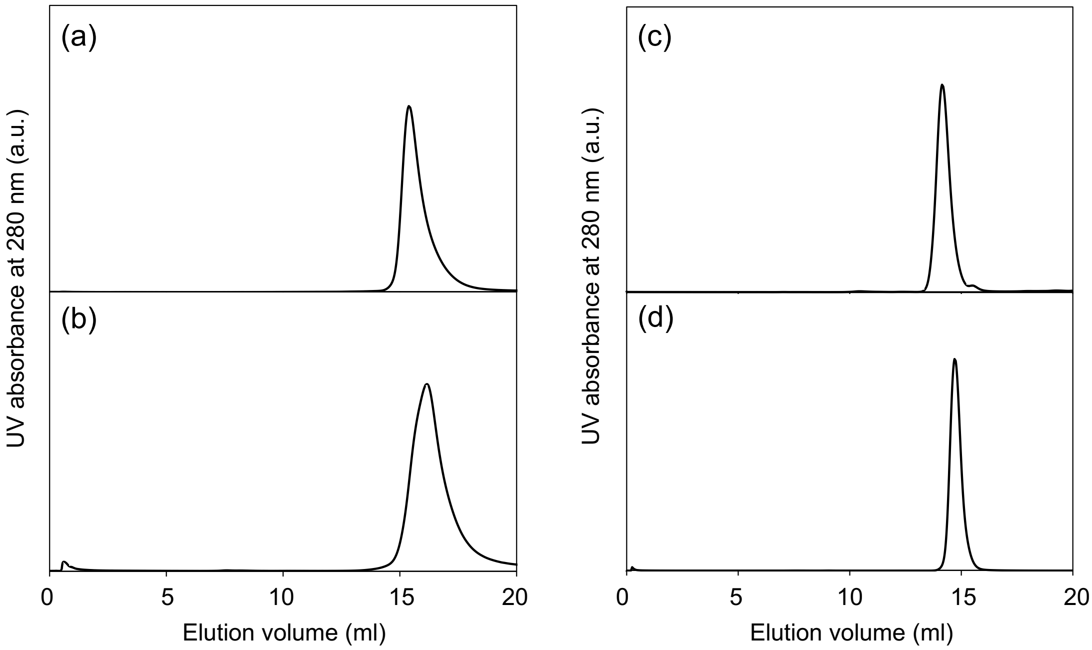

Supplementary Figure S3. Size-exclusion chromatograms of (a) IgG2a, (b) IgG2a(s), (c) C_L and (d)VHH. The experiments were conducted using a Superdex 200 10/300 GL column (GE Healthcare, Chicago, Illinois, USA) for IgG2a and IgG2a(s) and Superdex 75 10/300 GL column (GE Healthcare, Chicago, Illinois, USA) for C_L and VHH, which were equilibrated with phosphate-buffered saline (PBS) consisting of 137 mM NaCl, 2.7 mM KCl, 8.1 mM Na_2HPO_4, and KH_2PO_4 (pH 7.4) at room temperature.

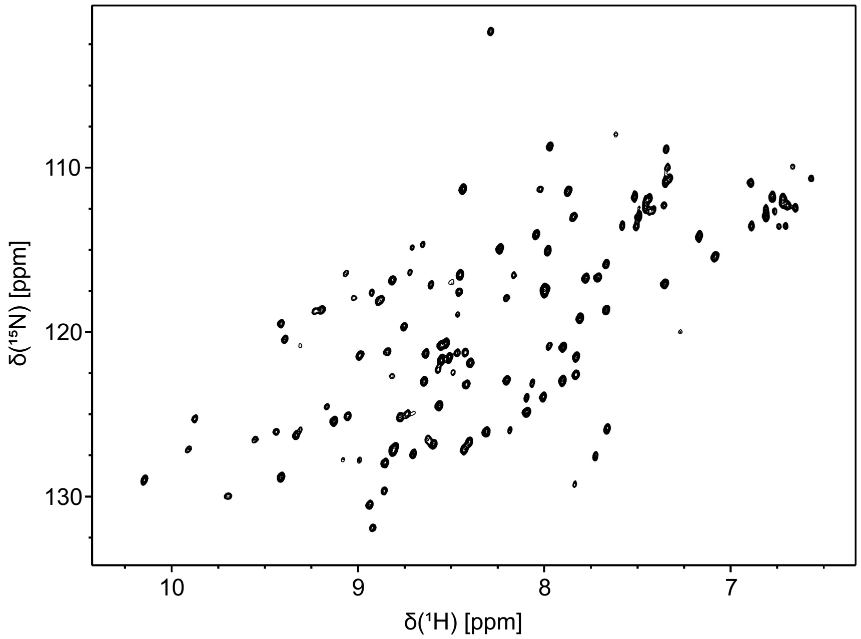

Supplementary Figure S4. ^{1}H-^{15}N HSQC spectrum of ^{15}N -labeled C_L recorded at 25°C. ^{15}N -labeled C_L was bacterially expressed using an M9 medium containing 0.5 g/L ^{15}N -labeled ammonium chloride according to a previously described protocol [30] and purified in the same way as for the unlabeled C_L protein. The protein was dissolved at a concentration of 600 μM in 0.25 ml of 5 mM sodium phosphate buffer (pH 6.0) containing 50 mM NaCl and 5% (v/v) D_2O. NMR spectral data were acquired at 25°C using an AVANCE 800 (Bruker BioSpin) spectrometer. Chemical shifts of ^{1}H were referenced to DSS (0 ppm), while ^{15}N chemical shifts were referenced indirectly using the gyromagnetic ratios of ^{15}N and ^{1}H ($\gamma^{15}N/\gamma^{1}H$ = 0.10132905).

Supplementary movie legends

Supplementary Movie S1: HS-AFM movie of C1

Captured at a scanning speed of 0.3 s/frame. Scan area: 50 nm × 50 nm. Pixel size: 50 × 50 pixels.

Supplementary Movie S2: HS-AFM movie of C1q

Captured at a scanning speed of 0.3 s/frame. Scan area: 50 nm × 50 nm. Pixel size: 50 × 50 pixels.

Supplementary Movie S3: HS-AFM movie of IgG2a

Captured at a scanning speed of 0.3 s/frame. Scan area: 40 nm × 40 nm. Pixel size: 62 × 62 pixels.

Supplementary Movie S4: HS-AFM movie of IgG2a(s)

Captured at a scanning speed of 0.3 s/frame. Scan area: 40 nm × 40 nm. Pixel size: 62 × 62 pixels.

Supplementary Movie S5: HS-AFM movie of IgG2a(s) interacting with C1

Captured at a scanning speed of 1 s/frame. Scan area: 149 nm × 149 nm. Pixel size: 57 × 57 pixels.

Supplementary Movie S6: HS-AFM movie of IgG2a(s) interacting with C1q

(a) Captured at a scanning speed of 1 s/frame. Scan area: 149 nm × 149 nm. Pixel size: 57 × 57 pixels. (b) Captured at a scanning speed of 0.15 s/frame. Scan area: 150 nm × 105 nm. Pixel size: 120 × 84 pixels.
